# Tricuspid Apparatus Detachment for Exposure of Ventricular Septal Defect: A Justified Approach?

**DOI:** 10.21470/1678-9741-2023-0202

**Published:** 2024-02-26

**Authors:** Rahul Bhushan, Vijay Grover

**Affiliations:** 1 Department of Cardiovascular and Thoracic Surgery, All India Institute of Medical Sciences Patna, Moti Nagar, Patna, India; 2 Department of Cardi ovascular and Thoracic Surgery, Dr Ram Manohar Lohia Hospital and Post Graduate Institute of Medical Education and Research, New Delhi, New Delhi, India

Dear Editor, we have read the original article by Çelik M et al.^[1]^ — “Comparison of Surgical Techniques Used in Ventricular Septal Defect Closure” — with profound interest. The article gives a detailed insight into surgical approaches and challenges faced for proper exposure of margin of ventricular septal defects (VSD). The authors did a commendable effort in reviewing their four-year experience with surgical closure of septal defects and made a comprehensive comparative analysis of different surgical approaches they have used, thus making a valuable contribution to literature.

However, there are multiple factors that need consideration and addressal in the given study. Repair of VSD is one of the commonest congenital cardiac surgeries performed globally, and trans right atrial (RA) approach is a globally accepted uniform norm owing to lesser complications and better outcomes in comparison to other approaches^[2]^. The authors in the given study second that and compare trans RA approach in three subgroups, *i.e.*, approach with retraction of tricuspid leaflet, tricuspid septal detachment (TSD) approach, and tricuspid chordal detachment (TCD) approach. The standard recommended technique is retraction of tricuspid leaflet while preserving native geometry of tricuspid apparatus, the TSD and TCD approach are reserved to circumstances with difficult VSD margin exposure and as a last resort to interfere with native tricuspid valvular apparatus^[2]^. The authors, in their study, have used a very high incidence of around 31% of patients undergoing TSD approach while 20.5% patients underwent TCD approach, summing up to more than half the cases, which is surprisingly high^[3]^. Even the index reference article by Pourmoghadam et al. in the study had used 31.5% of combined TSD and TCD approaches^[3]^. Also, the method adopted for specified surgical approach is illdefined and needs review if it was based on randomisation or on surgical requirement on case to case to basis, and if so, it would be prudent by the authors to mention guidelines/parameters used to adopt a given surgical approach.

Secondly, the authors used continuous suturing technique to close VSD. Though advocated by initial stalwarts of cardiac surgery, this approach makes vision of VSD margins a little difficult while interrupted suturing technique aids in vision by traction. How much did that play a role in need for excision of tricuspid valvular apparatus is a matter of debate. Also, usual cardiopulmonary bypass (CPB) time is quite short by using continuous suturing technique as a single suture in a running fashion, being quicker in comparison to using suture manager and interrupted pledged sutures one by one. Despite this, the CPB time of the study is on the longer side (83.2 minutes average), which is not comparable to other studies with continuous suturing technique for VSD closure and which offers a food of thought for justification of surgical intervention implied for visual aid while patient is exposed to risk of deleterious side effects of prolonged CPB time^[4]^. In the metanalysis by Yuan et al. scanning over database to pool 1,404 patients, tricuspid valve intervention was found to prolong CPB time (mean deviation [MD] = 7.75, *P*=.003) and cross-clamping time (MD = 7.77, *P<*.001) in a statistically significant way^[4]^.

In a prospective study of VSD closure by tricuspid valve detachment conducted by Lucchese et al. over 10 years, a total of 8.8% patients had moderate tricuspid regurgitation (TR) while 7.3% patients had severe TR^[5]^.These authors mention six (5.2%) patients having severe TR in preoperative echocardiographic evaluation while final analysis on follow-up showed that none of the patients enrolled had TR in the postoperative period. While the result is encouraging, it would have been prudent to shed a light on patients who preoperatively already had regurgitation across tricuspid valve and whether any concomitant tricuspid valve intervention in form of commissuroplasty was performed among those patients^[5]^.

Çelik M et al. have also included VSD closure performed as a combined procedure in addition to other cardiac defects namely arterial switch, interrupted aortic arch, etc. While this makes the case series unique in the fact that pure VSD is not an only inclusion, it introduces certain element of bias in the study since many times, in cases of arterial switch, a transpulmonary approach/trans aortic approach offers a better view of septal defect and a further intervention of detaching tricuspid valve leaflet/chordae might not be needed at all in the first place^[5]^.

Lastly, the authors have followed up patients only for one month for severity of TR progression. While immediate TR is common after repair of tricuspid apparatus post its detachment, late TR is also is a possible outcome. Thus, a longer follow-up and echocardiography evaluation are vital in coming to conclusion if routine use of septal detachment/chordal detachment shall be advocated for exposing VSD margin. It is a matter of great contention, and the step forward taken by the authors brings forth light on further research work needed on this common surgical practise of VSD closure and various possible surgical approaches needed to facilitate better view of margins, thus aiding in better surgical closure of septal defects.
